# Pathologic Stage of Nonsmall Cell Lung Cancer Patients Presenting as Resectable Cases After Neoadjuvant Therapy Did Not Predict the Prognosis

**DOI:** 10.1097/MD.0000000000001700

**Published:** 2015-10-09

**Authors:** Ching-Yang Wu, Jui-Ying Fu, Ching-Feng Wu, Yun-Hen Liu, Ming-Ju Hsieh, Yi-Cheng Wu, Cheng-Ta Yang, Ying-Huang Tsai

**Affiliations:** From the Division of Thoracic and Cardiovascular Surgery, Department of Surgery, Chang Gung Memorial Hospital, Chang Gung University, Taoyuan, Taiwan (C-YW, C-FW, Y-HL, M-JH, Y-CW); Division of Pulmonary and Critical Care, Department of Internal Medicine, Chang Gung Memorial Hospital, Chang Gung University, Taoyuan, Taiwan (J-YF, C-TY); and Division of Pulmonary and Critical Care, Department of Internal Medicine, Chang Gung Memorial Hospital, Chang Gung University, Chiayi, Taiwan (Y-HT).

## Abstract

According to the National Comprehensive Cancer Network (NCCN) guidelines, treatment plans for nonsmall cell lung cancer are to be based on cancer stage. Cancer staging for patients with resectable disease has been based on pathologic stage instead of preoperative clinical stage. However, the possibility of occult mediastinal lymph node metastases could lead to discrepancy between clinical and pathologic stage. While multi-modality treatments may be beneficial for patients with locally advanced disease, most studies have been based on clinical stage. The aim of this study was to identify the beneficial impact of neoadjuvant therapy and the prognostic value of final pathologic stage in these patients.

This study enrolled 530 lung cancer patients who received anatomic resection and mediastinal lymph node dissection at Chang Gung Memorial Hospital from January 2005 through June 2011. All resected specimens were examined by pathologists. Postoperative adjuvant therapies were given according to NCCN guideline recommendations. The clinico-pathologic factors of these patients were collected and analyzed.

Patients not receiving neoadjuvant therapy had a better probability of disease-free survival (*P* < 0.001) and overall survival (*P* = 0.0005), as well as a lower incidence of early relapse. Patients not receiving neoadjuvant therapy had a better disease-free survival rate in stages IA (*P* < 0.001), IB (*P* = 0.002), and IIB (*P* = 0.0117) from the point of view of final pathologic stage.

Patients receiving neoadjuvant therapy may experience a higher incidence of early relapse. Neoadjuvant therapy did not show definite benefits in the disease-free and overall survival rates from the point of view of final pathologic stage. Pathologic stage of nonsmall cell lung cancer patients who presented with resectable disease after neoadjuvant therapy did not predict the prognosis.

## INTRODUCTION

Lung cancer is a leading cause of death worldwide. In Taiwan, the incidence of death caused by lung cancer in 2012 was 25.5 deaths per 100,000 population.^1^ According to the National Comprehensive Cancer Network (NCCN) guidelines, the standard treatment modality in resectable nonsmall cell lung cancer is anatomic resection with mediastinal lymph node dissection.^2^ However, there is no consensus on managing patients with locally invasive nonsmall cell lung cancer. As the literature review shows, multi-modality treatments, including chemotherapy, radiation therapy, and surgery, may be beneficial for certain patients.^3–10^

Theoretically, neoadjuvant therapy should lead to decreasing tumor volume and control of possible systemic occult micro-metastases, while also facilitating the possibility of curative resection. However, debate about the survival benefit of neoadjuvant therapy remains.^11–13^ Most articles have analyzed this issue from the point of view of clinical stage, however, in clinical practice, further treatment plans for patients with resectable disease have been based on final pathologic stage instead of preoperative clinical stage. The literature review shows that discrepancies between clinical and pathologic stage are not uncommon and caused by the limitations of imaging tools. Agreement between the clinical and pathologic stages in the eras before and after positron emission tomography (PET) were 21.7% and 67.2%, respectively.^14–16^ With PET, small, low profile lesions or possible inflammations may cause false negative or positive findings.^17,18^ With computed tomography (CT), lesions <5 mm in diameter may not be seen because they could be hidden between 2 successive slices. Discrepancy between clinical staging and pathology may lead to inclusion of patients with less extensive disease and overestimation of survival benefit of neoadjuvant therapy. In order to eliminate possible overestimation of the efficacy of neoadjuvant therapy, we compared the survival status between patients receiving, and those not receiving neoadjuvant therapy, from the point of view of pathologic stage. The aim of this study was to identify the beneficial impact of neoadjuvant therapy and the prognostic value of final pathologic stage in patients presenting with resectable disease after preoperative neoadjuvant therapy.

## MATERIALS AND METHODS

### Patients

This study enrolled 605 lung cancer patients who received operations at Chang Gung Memorial Hospital from January 2005 through June 2011. Exclusion criteria included wedge resections due to poor pulmonary reserve (43 patients), small cell lung cancer patients (11 patients), patients who identified positive resection or those with pathologic III B or IV (25 patients). After these exclusions, only 530 patients were included in this study. Medical record data of these patients were retrospectively reviewed and all clinico-pathologic factors were collected for further survival analysis. The study was approved by the ethics committee of Chang Gung Memorial Hospital, under the Institutional Review Board number 103-5631B.

### Preoperative Evaluation

Patients received a chest CT scan that included the upper abdomen in order to identify the severity of their disease. Tissue proofing, including a bronchoscopic biopsy or a CT-guided biopsy, was arranged if the lesion was feasible. If no definite diagnosis was confirmed, a surgical biopsy was performed prior to anatomic resection in the same operation. Bone scan or PET was arranged in order to rule out possible distant metastases, except for brain metastases. A brain CT or magnetic resonance image (MRI) was arranged to rule out central nervous system metastases. Spirometry was also scheduled before the operation in order to identify the pulmonary reserve.

### Preoperative Treatment

For patients with resectable disease, no further preoperative therapy was given. For patients with advanced disease, neoadjuvant therapies, including chemotherapy, concurrent chemo-radiation therapy, or target therapy, were pursued according to the clinical situation. For clinical patients with N2 disease, 4 to 6 courses of cisplatin-based chemotherapy were given. For patients with locally advanced diseases, such as bulky N2 lymph node, clinical T4 invasive, or M1 disease, concurrent chemo-radiation therapy was arranged. Tyrosine kinase inhibitor (TKI) therapy was given to patients with unresectable disease who had a confirmed epidermal growth factor receptor (EGFR) mutation. Repeat staging was done after completion of the neoadjuvant therapy. Patients received anatomic resection and mediastinal lymph node dissection only when preoperative image survey revealed resectable disease. The cancer staging of all patients was done according to the American Joint Cancer Conference (AJCC) 7th TNM staging definitions.

### Operation, Postoperative Adjuvant Planning, and Surveillance Program

Anatomic resection, including lobectomy, bilobectomy, or pneumonectomy, was arranged depending on the severity of the disease via open thoracotomy or video-assisted thoracoscopic surgery (VATS). The pulmonary vein, artery, and bronchus were individually divided. Mediastinal lymph node dissection was then performed. All of the resected specimens, including tumors and mediastinal lymph nodes, were examined by pathologists. Postoperative adjuvant therapies were determined according to the final pathologic stage and the NCCN guideline recommendations. Patients were required to return to the outpatient department every 3 months, at which point a chest plain film or chest CT were utilized as image tools.

### Statistics

All collected clinico-pathologic factors were evaluated by univariate analysis. Categorical variables were compared using chi-squared tests, while continuous variables were compared using 2 sample *t* tests. Disease-free survival was defined as no evidence of relapse in the period from the date of the operation to the last follow-up date. Overall survival was defined as the period between the operation date and death. Relapse tumor tissue identified in the ipsilateral thoracic cage was defined as a local relapse. Pleural seeding or extra-pulmonary relapse was defined as a distant relapse. Disseminated relapse was defined as a combination of local and distant relapses. Survival status was represented with a Kaplan–Meier curve and compared using log-rank test. A *P* value <0.05 was considered statistically significant. All the analyses were performed using SAS, version 9 (SAS Institute, Cary, NC).

## RESULTS

This study included 530 lung cancer patients who received anatomic resection and mediastinal lymph node dissection at Chang Gung Memorial Hospital from January 2005 through June 2011, 52.3% of whom were female. The mean age of all patients was 62.28 years old. The mean tumor size was 3.19 cm and the majority (70.4%) of the cell types was adenocarcinoma. The mean dissected mediastinal lymph node number was 18.1 while the mean metastatic lymph node number was 1.01; 60.4% of the patients received anatomic resection and mediastinal lymph node dissection via thoracotomy. Patients’ characteristics are listed in Table 1. We further compared patients receiving or not receiving neoadjuvant therapy and their characteristics, as listed in Table 2. Differences in microscopic presentation, including tumor cell type (*P* = 0.01), differentiation grade (*P* < 0.0001), visceral pleural invasion (*P* = 0.0002), angiolymphatic invasion (*P* = 0.0003), perineural invasion (*P* = 0.001), tumor necrosis (*P* < 0.0001), lymphocytic infiltrates (*P* < 0.0001), and mitosis (*P* = 0.02) were identified among these 2 groups. The comparison of tumor size and the mediastinal lymph node status revealed no differences between the 2 groups. However, the final distribution of pathologic staging did reflect a difference between these 2 groups (*P* < 0.001).

**TABLE 1:**
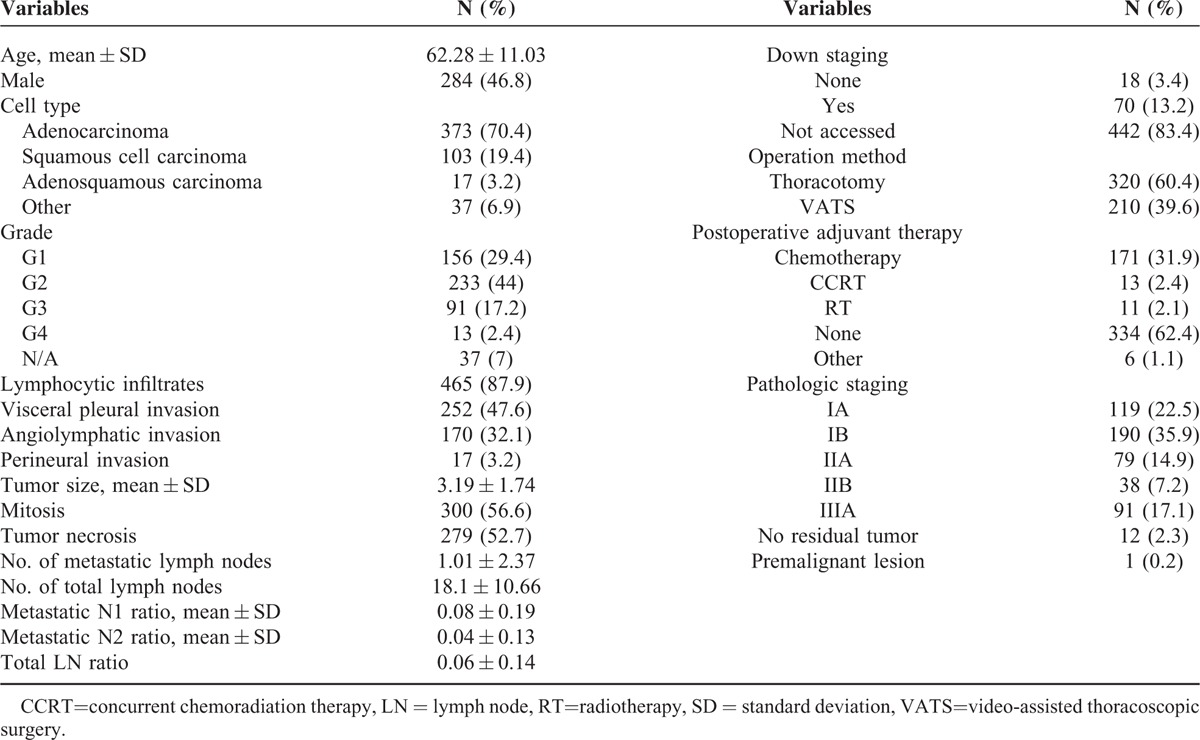
Patients’ Characteristics

**TABLE 2:**
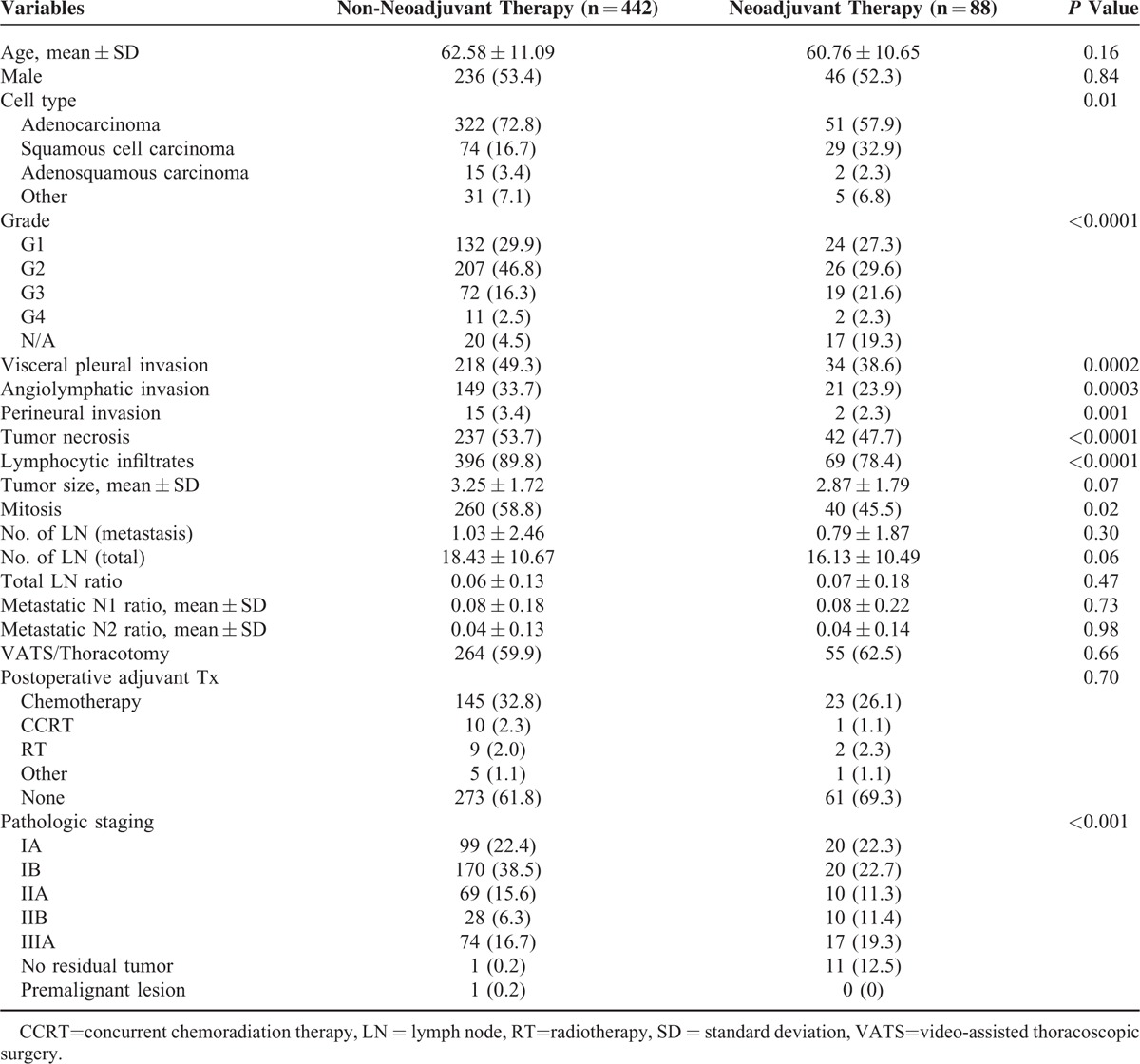
Comparison Between Non-Neoadjuvant Therapy and Neoadjuvant Therapy Groups

**FIGURE 1:**
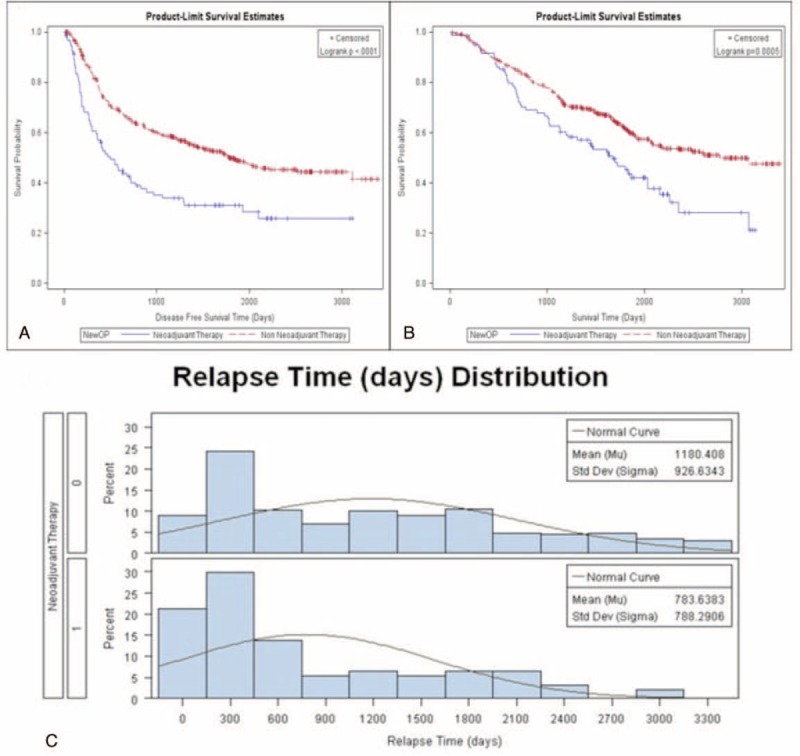
(A) Disease-free survival of all patients (neoadjuvant group vs non-neoadjuvant group). (B) Overall survival of all patients (neoadjuvant group vs non-neoadjuvant group). (C) Relapse pattern of all patients.

**FIGURE 2:**
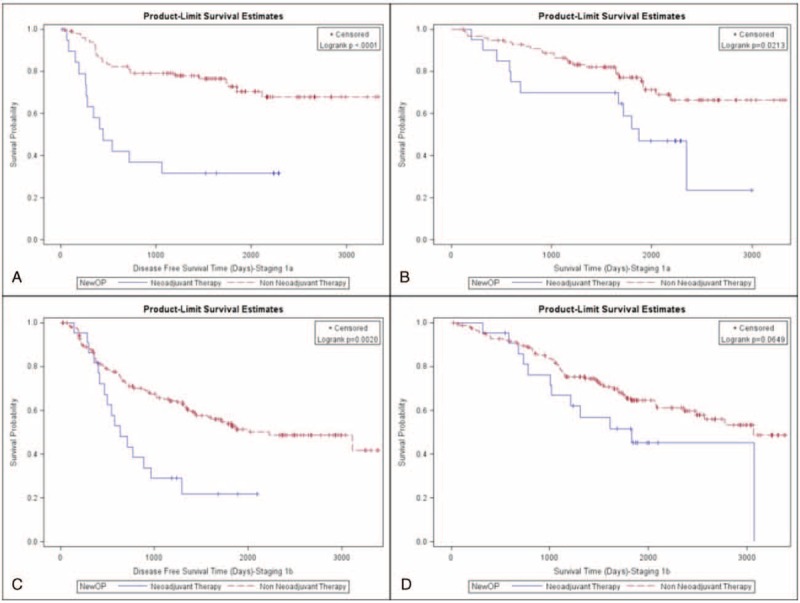
(A) Disease-free survival of pathologic stage IA patients (neoadjuvant group vs non-neoadjuvant group). (B) Overall survival of pathologic stage IA patients (neoadjuvant group vs non-neoadjuvant group). (C) Disease-free survival of pathologic IB patients (neoadjuvant group vs non-neoadjuvant group). (D) Overall survival of pathologic IB patients (neoadjuvant group vs non-neoadjuvant group).

**FIGURE 3:**
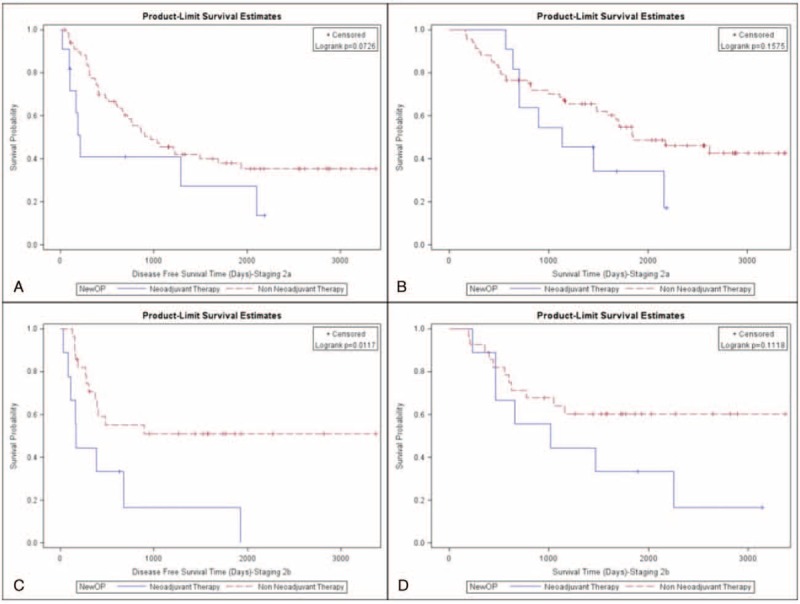
(A) Disease-free survival of pathologic stage IIA patients (neoadjuvant group vs non-neoadjuvant group). (B) Overall survival of pathologic stage IIA patients (neoadjuvant group vs non-neoadjuvant group). (C) Disease-free survival of pathologic IIB patients (neoadjuvant group vs non-neoadjuvant group). (D) Overall survival of pathologic IIB patients (neoadjuvant group vs non-neoadjuvant group).

**FIGURE 4:**
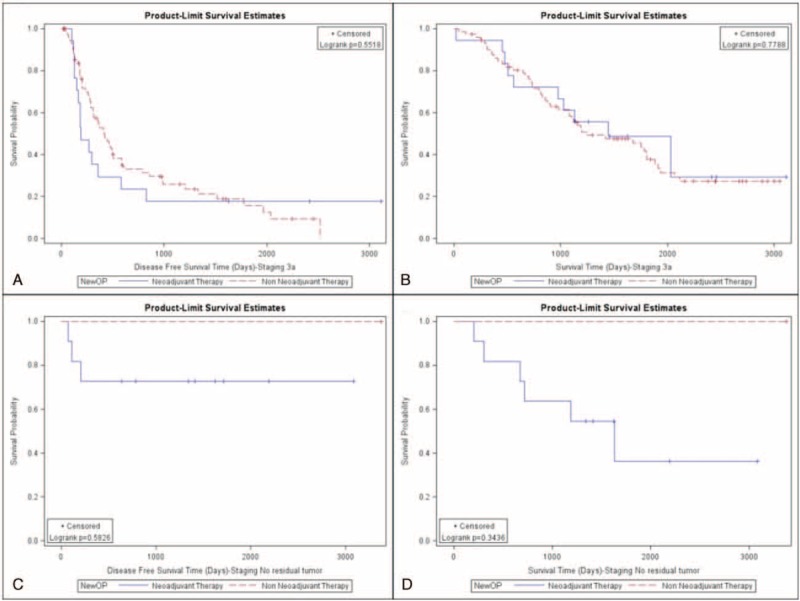
(A) Disease-free survival of pathologic stage IIIA patients (neoadjuvant group vs non-neoadjuvant group). (B) Overall survival of pathologic stage IIIA patients (neoadjuvant group vs non-neoadjuvant group). (C) Disease-free survival of no residual tumor patients (neoadjuvant group vs non-neoadjuvant group). (D) Overall survival of no residual tumor patients (neoadjuvant group vs non-neoadjuvant group).

Both disease-free survival and overall survival of these 2 groups were further analyzed. Patients not receiving neoadjuvant therapy had better disease-free (48% vs 32%; *P* < 0.001; Fig. 1A) and overall survival rates (60% vs 44%; *P* = 0.0005; Fig. 1B). We further clarify that patients not receiving neoadjuvant therapy had a better disease-free survival rate in pathologic stage IA (Fig. 2A, *P* < 0.001), IB (Fig. 2C, *P* = 0.002), and IIB (Fig. 3C, *P* = 0.0117). However, the difference of disease-free survival between patient with and without neoadjuvant therapy did not identify in pathologic stage IIA (Fig. 3A, *P* = 0.0726), IIIA (Fig. 4A, *P* = 0.5518), and with no residual tumor (Fig. 4C, *P* = 0.5826). In addition, the cumulative overall survival curve showed neoadjuvant therapy did not show definite survival benefits in the overall survival in any pathologic stage (Figs. 2B and D, 3B and C, and 4B and C). Patients who received neoadjuvant therapy had a high percentage (20% vs 10%) of early relapse compared with those who not receiving neoadjuvant therapy (Fig. 1C). We further analyzed the relapse pattern between these 2 groups and found that patients receiving neoadjuvant therapy also had a higher percentage of local (*P* < 0.0003), distant, (*P* = 0.001), and disseminated relapse (*P* < 0.0007) compared with those who did not receive neoadjuvant therapy (Table 3).

**TABLE 3:**
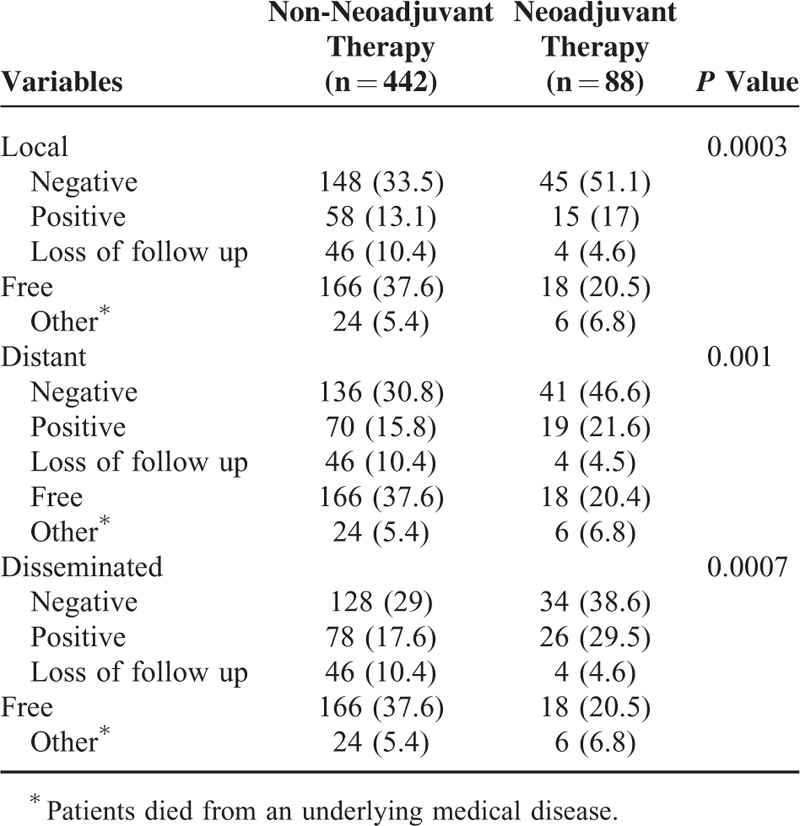
Comparison of Relapse Pattern Between the Non-Neoadjuvant and Neoadjuvant Group

## DISCUSSION

In our study, we found that patients receiving neoadjuvant therapy had worse disease-free survival rates than those who not receiving neoadjuvant therapy in pathologic stages IA, IB, and IIB. This may have been due to occult micro-metastases that could not be ruled out despite detailed image examination. The neoadjuvant group with pathologic stage IIA had a poor disease survival trend (*P* = 0.0726) compared with those not receiving neoadjuvant therapy. This result may be related to the relatively small case number and further investigation is therefore warranted. The complete pathologic response in our study was 12.5% (11/88), similar to the reported complete pathologic response in the literature, ranging from 3.7% to 10.5%.^19,20^ For patients with no residual tumor, clinical scenarios differed between those not receiving neoadjuvant therapy and those receiving it. In the former, all of the tumor tissue was excised during an intra-operative biopsy or from a premalignant lesion while the latter all tumor cells were presumed eradicated by neoadjuvant therapy. Both groups presented with no tumor tissue in the final resected specimens, which is why we compared these 2 groups at the same basis from a pathologic point of view. There is no statistical difference in the disease-free survival rate between both groups. From the literature review, the complete pathologic response of the neoadjuvant group may be correlated to disease-free survival.^21,22^ Our findings imply that the complete pathologic response of the group receiving neoadjuvant therapy may have similar disease-free survival rates to patients presenting with the earliest stages of lung cancer, including tiny tumors without mediastinal lymph node invasion and premalignant change. However, further investigation is warranted because of the limited case numbers. For pathologic stage IIIA, the 2 groups showed no difference in disease-free survival. This result may be related to the use of the same therapy modalities despite differing treatment sequences in both groups.

With regard to overall survival, we were only able to identify that patients with pathologic stage IA showed a statistically significant difference between the non-neoadjuvant and neoadjuvant groups (Fig. 2B, *P* = 0.02). As we know, the possibility of occult micro-metastases cannot be completely ruled out and they may be encountered with higher probability in patients receiving neoadjuvant therapy. Furthermore, no adjuvant therapy was given for patients with pathologic stage IA, eliminating the survival impact of the adjuvant therapy and truly revealing the natural disease course in the 2 groups. Our results showed that even if neoadjuvant therapy leads down to stage IA in the final pathologic examination, aggressive maintenance therapy is still recommended because of worse overall survival rate. For pathologic stage IB, a worse overall survival trend (Fig. 2D, *P* = 0.0649) was identified but this still needs further investigation. For patients with pathologic stage IIA (*P* = 0.1575), IIB (*P* = 0.1118), and IIIA (*P* = 0.7788), overall survival showed no statistical difference between the 2 groups. For these patients, more extensive disease status was established preoperatively and different type of adjuvant therapy was given. Further analysis is warranted. For patients without residual tumor in the resected specimen, the overall survival rate showed no statistical difference (*P* = 0.3436) between those receiving neoadjuvant therapy and those not receiving it. Further investigation is warranted because of limited cases.

In our study, we found that characteristics between the non-neoadjuvant and neoadjuvant group were totally different. Patients not receiving neoadjuvant therapy had better disease-free (48% vs 32%; *P* < 0.001; Fig. 1) and overall survival rates (60% vs 44%; *P* = 0.0005; Fig. 1). This showed that neoadjuvant therapy did not provide a definite benefit in overall survival from the view of the final pathologic stage obtained from the resected specimen. In addition, patients receiving neoadjuvant therapy had a high percentage (20% vs 10%) of early relapse compared with those who not receiving neoadjuvant therapy (Fig. 1C). Furthermore, patients with neoadjuvant therapy had a higher rate of local (*P* < 0.0003), distant (*P* = 0.001), and disseminated relapse (*P* < 0.0007) compared with those without neoadjuvant therapy.

This may be caused by possible residual tumor cells existing in the previous tumor invasion area and occult micrometastases. Our study result showed that pathologic stage of nonsmall cell lung cancer patients who presented with resectable disease after neoadjuvant therapy did not predict the prognosis. For patients who received treatment with neoadjuvant therapy and presented with resectable disease, even if pathologic examination identified a disease at a lower stage, aggressive adjuvant treatment and surveillance are still recommended due to the high risk of disease recurrence.

This study had some limitations. First, this is a retrospective study with a medium amount of cases. Most articles have analyzed this issue from the view of the clinical stage and so the survival benefit of neoadjuvant therapy may have been overestimated. However, our study differs from such previous literature in that we analyze the issue from the pathologic stage view, the basis of further therapeutic planning after operation. Our findings suggest more aggressive treatment for these patients because of the risk of occult metastases. Second, the agreement between clinical and pathologic staging in our study was 45.1% (239/530). Several reasons led to this discrepancy, including insurance policy and limitations of image tools and specimen preparation. In Taiwan, PET has no longer been available since 2006 because of national health insurance restrictions. In addition, chest tomography could not identify visceral pleural invasion or distinguish between the consolidation and tumor parts of a lesion. Furthermore, the fixation preparation may lead to tumor shrinkage, which also leads to inconsistencies between the clinical and pathologic stage.^23^ Therefore, we decided to compare survival results between patients with and without neoadjuvant therapy from the point of view of the pathologic stage. The consistency between clinical and pathologic stage in this study remains within acceptable percentage range when compared with literature results. Third, we used chemotherapy, chemo-radiation, or TKI therapy as the neoadjuvant treatments according to the patients’ individual clinical scenarios. Because of limited case numbers in each treatment modality, we could not further analyze the difference among these modalities.

Although these limitations remain, our study had following important findings. In this study, patients receiving neoadjuvant therapy who had residual diseases not only had a worse survival rate but also a higher incidence of early relapse compared with those not receiving neoadjuvant therapy. This may have been due to residual tumor cells existing in the previous tumor invasion area and occult micrometastases. More aggressive postoperative adjuvant therapy and a closer follow-up program are recommended for neoadjuvant therapy patients. In addition, we also identified that there was no difference between neoadjuvant therapy patients with complete pathologic response and those with tiny lesions and premalignant lesion. This finding may imply that neoadjuvant patients with complete pathologic response had similar survival status with those presenting as the earliest stage of disease; however, further investigation is required to verify this, due to limited case numbers.

## CONCLUSION

Patients with a locally advanced disease who received neoadjuvant therapy completely differed from those who did not receive neoadjuvant therapy. Patients who received neoadjuvant therapy may experience a higher incidence of early relapse and aggressive adjuvant treatment and surveillance are still recommended.

Neoadjuvant therapy did not show definite benefits in the disease-free and overall survival rates from the view of the final pathologic stage. Pathologic stage of nonsmall cell lung cancer patients who presented with resectable disease after neoadjuvant therapy did not predict the prognosis.

## References

[1] Department of Statistics, Ministry of Health Welfare, Taiwan. Analysis of Major cause of death in year 2012 ( MK101) p.17 http://www.mohw.gov.tw/cht/DOS/Statistic.aspx?f_list_no=312&fod_list_no=2747.

[2] National Comprehensive Cancer Network Guideline Version 7. 2015 Principle of surgical therapy NSCL-B 1-4. http://www.nccn.org/professionals/physician_gls/f_guidelines.asp.

[3] Koshy M Fedewa SA Malik R Improved survival associated with neoadjuvant chemoradiation in patients with clinical stage IIIA(N2) non-small-cell lung cancer. *J Thorac Oncol* 2013; 8:915–922. 23608815 10.1097/JTO.0b013e31828f68b4

[4] Betticher DC Hsu Schmitz SF Tötsch M Mediastinal lymph node clearance after docetaxel-cisplatin neoadjuvant chemotherapy is prognostic of survival in patients with stage IIIA pN2 non-small-cell lung cancer: a multicenter phase II trial. *J Clin Oncol* 2003; 21:1752–1759. 12721251 10.1200/JCO.2003.11.040

[5] Rosell R Gómez-Codina J Camps C A randomized trial comparing preoperative chemotherapy plus surgery with surgery alone in patients with non-small-cell lung cancer. *N Engl J Med* 1994; 330:153–158. 8043059 10.1056/NEJM199401203300301

[6] Roth JA Fossella F Komaki R A randomized trial comparing perioperative chemotherapy and surgery with surgery alone in resectable stage IIIA non-small-cell lung cancer. *J Natl Cancer Inst* 1994; 86:673–680. 8158698 10.1093/jnci/86.9.673

[7] Burdett S Stewart LA Rydzewska L A systematic review and meta-analysis of the literature: chemotherapy and surgery versus surgery alone in non-small cell lung cancer. *J Thorac Oncol* 2006; 1:611–621. 17409927

[8] Berghmans T Paesmans M Meert AP Survival improvement in resectable non-small cell lung cancer with (neo)adjuvant chemotherapy: results of a meta-analysis of the literature. *Lung Cancer* 2005; 49:13–23. 15949586 10.1016/j.lungcan.2005.01.002

[9] Depierre A Milleron B Moro-Sibilot D Preoperative chemotherapy followed by surgery compared with primary surgery in resectable stage I (except T1N0), II, and IIIa non-small-cell lung cancer. *J Clin Oncol* 2002; 20:247–253. 11773176 10.1200/JCO.2002.20.1.247

[10] Pisters KM Vallières E Crowley JJ Surgery with or without preoperative paclitaxel and carboplatin in early-stage non-small-cell lung cancer: Southwest Oncology Group Trial S9900, an intergroup, randomized, phase III trial. *J Clin Oncol* 2010; 28:1843–1849. 20231678 10.1200/JCO.2009.26.1685 PMC2860367

[11] Detterbeck FC Boffa DJ Tanoue LT The new lung cancer staging system. *Chest* 2009; 36:260–271. 19584208 10.1378/chest.08-0978

[12] Song W-A Zhou N-K Wang W Survival benefit of neoadjuvant chemotherapy in non-small cell lung cancer. An updated meta-analysis of 13 randomized control trials. *J Thorac Oncol* 2010; 5:510–516. 20107424 10.1097/JTO.0b013e3181cd3345

[13] Burdett S Rydzewska LH Tierney JF Preoperative chemotherapy for non-small-cell lung cancer: a systematic review and meta-analysis of individual participant data. *Lancet* 2014; 383:1561–1571. 24576776 10.1016/S0140-6736(13)62159-5 PMC4022989

[14] Fernandes G Sucena M Lombardia E Non small cell lung cancer—comparison between clinical and pathological staging. *Rev Port Pneumol* 2006; 12:337–357. 16969567

[15] McCann J PET scans approved for detecting metastatic non-small-cell lung cancer. *J Natl Cancer Inst* 1998; 21:94–96.90. 9450567 10.1093/jnci/90.2.94

[16] Vaz AP Fernandes G Souto Moura C Integrated PET/CT in non small cell lung cancer staging—clinical and pathological agreement. *Rev Port Pneumol* 2012; 18:109–114. 22405953 10.1016/j.rppneu.2012.01.004

[17] Chiu CH Yeh YC Lin KH Histological subtypes of lung adenocarcinoma have differential ^18^F-fluorodeoxyglucose uptakes on the positron emission tomography/computed tomography scan. *J Thorac Oncol* 2011; 6:1697–1703. 21869716 10.1097/JTO.0b013e318226b677

[18] Poettgen C Theegarten D Eberhardt W Correlation of PET/CT findings and histopathology after neoadjuvant therapy in non-small cell lung cancer. *Oncology* 2007; 73:316–323. 18497503 10.1159/000134474

[19] Rosell R Gómez-Codina J Camps C Preresectional chemotherapy in stage IIIA non-small-cell lung cancer: a 7-year assessment of a randomized controlled trial. *Lung Cancer* 1999; 26:7–14. 10574676 10.1016/s0169-5002(99)00045-8

[20] Felip E Rosell R Maestre JA Preoperative chemotherapy plus surgery versus surgery plus adjuvant chemotherapy versus surgery alone in early-stage non-small-cell lung cancer. *J Clin Oncol* 2010; 28:3138–3145. 20516435 10.1200/JCO.2009.27.6204

[21] Mouillet G Monnet E Milleron B Intergroupe Francophone de Cancérologie Thoracique (IFCT) Pathologic complete response to preoperative chemotherapy predicts cure in early-stage non-small-cell lung cancer: combined analysis of two IFCT randomized trials. *J Thorac Oncol* 2012; 7:841–849. 22722786 10.1097/JTO.0b013e31824c7d92

[22] Milleron B Westeel V Quoix E Complete response following preoperative chemotherapy for resectable non-small cell lung cancer: accuracy of clinical assessment using the French trial database. *Chest* 2005; 128:1442–1447. 16162741 10.1378/chest.128.3.1442

[23] Hsu PK Huang HC Hsieh CC Effect of formalin fixation on tumor size determination in stage I non-small cell lung cancer. *Ann Thorac Surg* 2007; 84:1825–1829. 18036892 10.1016/j.athoracsur.2007.07.016

