# Pathologic Stage of Nonsmall Cell Lung Cancer Patients Presenting as Resectable Cases After Neoadjuvant Therapy Did Not Predict the Prognosis: Erratum

**DOI:** 10.1097/01.md.0000499790.86340.f9

**Published:** 2015-10-30

In the article “Pathologic Stage of Nonsmall Cell Lung Cancer Patients Presenting as Resectable Cases After Neoadjuvant Therapy Did Not Predict the Prognosis^1^, which appeared in Volume 94, Issue 40 of *Medicine*, the total number of patients enrolled in the study should have been stated as 609, not 605, as was stated in the Materials and Methods section. The article has since been corrected online.
